# Retroperitoneal laparoscopic partial adrenalectomy (RLPA) for 20-40 mm nonfunctional adrenal tumors in the day surgery mode

**DOI:** 10.3389/fendo.2022.1099818

**Published:** 2022-12-20

**Authors:** Xuwen Li, Haibo Xi, Yue Yu, Wei Liu, Xiaoping Zhu, Zhixian Gong, Bin Fu, Gongxian Wang, Xiaochen Zhou

**Affiliations:** ^1^ Department of Urology, The First Affiliated Hospital of Nanchang University, Nanchang, China; ^2^ Department of Day Ward, The First Affiliated Hospital of Nanchang University, Nanchang, China

**Keywords:** adrenal tumor, retroperitoneal laparoscopic partial adrenalectomy, day surgery mode, hospital stay, hospitalization cost

## Abstract

**Objectives:**

To investigate the outcome and safety of retroperitoneal laparoscopic partial adrenalectomy in the treatment of nonfunctional unilateral adrenal tumors in the day surgery mode.

**Methods:**

Nineteen patients bearing nonfunctional unilateral 20-40 mm adrenal tumors were prospectively enrolled and underwent retroperitoneal laparoscopic partial adrenalectomy in the day surgery unit of our hospital between June 2021 and March 2022. All patients were diagnosed with non-functional adrenal tumors as outpatients before being admitted to the day surgery unit with their consent. Patient demographics and perioperative data were prospectively documented. The patients were followed up by telephone on day 1, 3 and 7 after discharge and followed up for 6 months.

**Results:**

The patient’s age was 50.5 ± 11.9 yr (range from 19.0 - 69.0). Seven patients were female. Twelve patients underwent surgery on the left side. The maximal diameter of tumor was 28.3 ± 5.7 mm (20.0 - 40.0 mm). Operation time was 72.1 ± 14.9 min (58.0 - 120.0 min). Mean blood loss was 64.7 ± 50.4 ml (30.0 - 200.0 ml). The gastrointestinal function recovery time was 9.7 ± 2.6 h (6.0 - 16.0 h). Retroperitoneal drainage was removed 24.8 ± 13.3 h (range 18.0 - 72.0) after surgery. Four patients were transferred to the general ward for postoperative management, while others were discharged within 24 hours after surgery. Length of hospital stay was 48.8 ± 13.1 h (38.0 - 85.0h). Hospitalization expense was 24168.4 ± 2910.3 RMB¥ (20844.3 - 34369.8 RMB¥). Postoperative pathology revealed 17 cortical adenoma, 1 pheochromocytoma and 1 lymphatic duct tumor.

**Conclusion:**

Retroperitoneal laparoscopic partial adrenalectomy for nonfunctional unilateral adrenal tumors in the day surgery mode is safe when strict selection criteria and perioperative management protocol are followed, which has the potential to shorten length of hospital stay and reduce lower hospitalization costs.

## 1 Introduction

Adrenal tumors are common, affecting 8.7% of the population (1). Although most adrenal tumors are benign, the management protocol of adrenal tumors always requires thorough urological and endocrinological studies due to their potential to overproduce hormones that affect organs throughout the body (2). Surgical excision of adrenal tumors ≥ 20 mm has become a consensus among urologists (3). Retroperitoneal laparoscopic adrenalectomy (RLA) is currently the first-line choice to remove benign adrenal tumors (4, 5). However, a growing number of researchers believe that for small (20-40 mm) adrenal tumors, it is preferable to preserve some healthy adrenal tissue in the aim of lowering the risk of postoperative adrenal insufficiency (6, 7).

Day surgery mode is a new surgery management mode proposed by the famous British surgeon Nicoll (8, 9), which means that within 24 h, patients who meet the inclusion criteria are arranged to complete the admission, operation and discharge in a pre-planned protocol. Under special circumstances, patients with delayed hospitalization should not stay longer than 48 hours. “Day surgery mode” is a treatment mode favored by surgeons in Europe and America because it can significantly reduce the length of hospital stay for patients and effectively make use of scarce medical resources (10). In our country, day surgery management mode is in the stage of development and exploration for many types of surgical interventions, including adrenalectomy.

At present, RLA in day surgery mode has been reported in some developed countries and large hospitals in China (11, 12). However, due to the potential bleeding risk of partial adrenalectomy, there have been no reports of retroperitoneal laparoscopic partial adrenalectomy (RLPA) in this mode. As such, we conducted this study to investigate the efficacy and safety of RLPA in the treatment of 20-40 mm nonfunctional unilateral adrenal tumors in the day surgery mode.

## 2 Materials and methods

The study was approval of by institutional review board and ethics committee of the First Affiliated Hospital of Nanchang University. Documentation of medical history, laboratory studies and non-contrast/contrast enhanced adrenal CT were performed prior to administration in the day surgery unit. Patients bearing non-functional unilateral adrenal tumor ranged from 20 to 40mm were enrolled after multidisciplinary discussion of urologists, endocrinologists, and anesthesiologists. Patients with ASA score > 2, younger than 18 yr or older than 70 yr were excluded. Prior to surgery, all candidates were re-evaluated and informed of the surgical and perioperative management plan by a urologist. A signed consent form was obtained from each candidate.

All surgeries were performed by the same experienced urologist. Patient was anesthetized and placed on flank position for a standard retroperitoneal laparoscopic partial adrenalectomy. Only the tumor was removed and hemostasis was achieved by a combination of bipolar and hem-o-locs. A drainage tube was placed through a trocar site at the end of surgery. Two hundred mg hydrocortisone was given during surgery to minimize the risk of adrenal crisis. On the first and second day after surgery, 100 mg and 50 mg hydrocortisone were given, respectively for the same reason.

Postoperative vital signs were closely monitored and postoperative rehabilitation education was conducted. Patients were encouraged to ambulate as soon as possible. The recovery time of gastrointestinal function and the status of drainage fluid were observed. Urethral catheter was removed 8 hours after surgery and drainage tube was removed when discharge was negligible or < 20 ml. Patients were discharged after all tubes were removed. Patients with inconformity or drainage tube not removed within 48h were then transferred to the general ward for further management. All patients were followed up by telephone 1,3 and 7 days after discharge. All patients were followed up for at least 6 months.

Patient demographic and perioperative data were prospectively documented, including tumor size, laterality, operation time, blood loss, gastrointestinal function recovery time, drainage tube placement time, complications, hospital stay and hospitalization expense were recorded. The operation time was defined as the time from first incision to all incisions were closed. Gastrointestinal function recovery time was defined as the time between leaving the operating room and the first passage of gas or defecation.

Quantitative variables were expressed as mean ± SD and qualitative variables were expressed as categorical variables (i.e., yes or no). SPSS V25.0 software was used to perform all the statistical analyses.

## 3 Results

During June 2021 to March 2022, 19 patients were enrolled. The demographic and preoperative data were summarized in **Table 1**. The patient’s age (mean ± SD) was 50.5 ± 11.9 yr (19.0 - 69.0). Seven patients were female. Mean BMI was 24.4 ± 3.6 kg/m^2^ (17.8 - 33.9). Twelve patients underwent surgery on the left side. The tumor maximal diameter was 28.3 ± 5.7 mm (20.0 - 40.0).

**Table 1:** Demographics and preoperative data.

	n = 19
Age (yr), mean (SD), range	50.5 (11.9), 19.0 - 69.0
Weight (kg), mean (SD), range	67.6 (13.9), 41.0 - 93.0
BMI (kg/m^2^), mean (SD), range	24.4 (3.6), 17.8 - 33.9
Gender, n (%)	
Female	7 (36.8%)
Male	12 (63.2%)
Laterality, n (%)	
L	12 (63.2%)
R	7 (36.8%)
Tumor maximal diameter (mm), mean (SD), range	28.3 (5.7), 20.0 - 40.0

Laparoscopic partial adrenalectomy was performed in all patients successfully. As summarized in **Table 2**, the operation time was 72.1 ± 14.9 min (58.0 - 120.0). Mean blood loss was 64.7 ± 50.4 ml (30.0 - 200.0). The gastrointestinal function recovery time was 9.7 ± 2.6 h 6.0 - 16.0), and the drainage tube placement time was 24.8 ± 13.3 h (18.0 - 72.0). Four patients were transferred to the general inpatient unit for extended hospitalization: one patient suffered from postoperative fever, two patients had prolonged drainage, and one patient needed longer observation for concerning vitals. Despite the delay, these 4 patients were all discharged within 85 h. The mean Hospital stay was 48.8 ± 13.05 h (38.0 - 85.0). And hospitalization expense was 24168.4 ± 2910.25 RMB¥ (20844.3 - 34369.8). Pathological study revealed 17 cortical adenomas, 1 pheochromocytoma and 1 lymphatic duct tumor. Telephone follow-up on days 1,3 and 7 after discharge were performed and found no significant concerns. Other complications were not noted during a minimal follow-up of 6 month.

**Table 2:** Intraoperative and postoperative data.

	n = 19
Operation time (min), mean (SD), range	72.1 (14.9), 58.0 - 120.0
Blood loss (ml), mean (SD), range	64.7 (50.4), 30.0 - 200.0
Gastrointestinal function recovery time (h), mean (SD), range	9.7 (2.6), 6.0 - 16.0
Drainage tube placement time (h), mean (SD), range	24.8 (13.3), 18.0 - 72.0
Transfer to general ward, n (%)	4 (21.1%)
Reasons for changing wards, n (%)	
Fever	1 (5.3%)
Not meet extubation standards	2 (10.5%)
Watchful waiting	1 (5.3%)
Hospital stay (h), mean (SD), range	48.8 (13.0), 38.0 - 85.0
Hospitalization expense (RMB¥), mean (SD), range	24168.4 (2910.3), 20844.3 - 34369.8
Pathologic, n (%)	
Cortical adenomas	16 (84.2%)
Pheochromocytoma	2 (10.5%)
Lymphatic duct tumor	1 (5.3%)

After removing the 4 delayed discharged patients from the 19-patient data set, we analyzed the 15 patients who were discharged following day surgery mode protocol again and summarized the data in **Table 3**, **4**. The patient’s age was 50.1 ± 13.0 yr (29.0.0 - 69.0). Six patients were female. Mean BMI was 24.5 ± 3.7 kg/m^2^ (17.8 - 33.9). Ten patients underwent surgery on the left side. The tumor maximal diameter was 25.9 ± 3.7 mm (20.0 - 32.0). The operation time was 66.1 ± 5.0 min (58.0 - 75.0). Mean blood loss was 45.3 ± 11.9 ml (30.0 - 70.0). The gastrointestinal function recovery time was 9.3 ± 2.6 h (6.0 - 16.0). Drainage tube placement time was 20.0 ± 2.3 h (18.0 - 24.0). The mean Hospital stay was 42.9 ± 2.6 h (38.0 - 48.0). Hospitalization expense was 23097.7 ± 1171.12 RMB¥ (20844.3 - 24832.5). Pathological study revealed all tumors were cortical adenomas.

**Table 3:** Demographics and preoperative data.

	n = 15
Age (yr), mean (SD), range	50.1 (13.0), 29.0 - 69.0
Weight (kg), mean (SD), range	67.2 (13.8), 41.0 - 93.0
BMI (kg/m^2^), mean (SD), range	24.5 (3.7), 17.8 - 33.9
Gender, n (%)	
Female	6 (40.0%)
Male	9 (50.0%)
Laterality, n (%)	
L	10 (66.7%)
R	5 (33.3%)
Tumor maximal diameter (mm), mean (SD), range	25.9 (3.7), 20.0 - 32.0

**Table 4:** Intraoperative and postoperative data.

	n = 15
Operation time (min), mean (SD), range	66.1 (5.0), 58.0 - 75.0
Blood loss (ml), mean (SD), range	45.3 (11.9), 30.0 - 70.0
Gastrointestinal function recovery time (h), mean (SD), range	9.3 (2.6), 6.0 - 16.0
Drainage tube placement time (h), mean (SD), range	20.0 (2.3), 18.0 - 24.0
Transfer to general ward, n (%)	0 (0%)
Reasons for changing wards, n (%)	
Fever	–
Drainage of fluid more	–
Watchful waiting	–
Hospital stay (h), mean (SD), range	42.9 (2.6), 38.0 - 48.0
Hospitalization expense (RMB¥), mean (SD), range	23097.7 (1171.1), 20844.3 - 24832.5
Pathologic, n (%)	
Adenoma sebaceum	15 (100.0%)
Pheochromocytoma	0 (0%)
Lymphatic duct tumor	0 (0%)

## 4 Discussion

With the development of the concept of minimally invasive surgery, the surgical treatment of adrenal tumors has evolved from open surgery to laparoscopic surgery, from total resection to partial resection. Michael Gagner performed the first laparoscopic resection of the adrenal gland in 1992 (13). Since then, the technique has been perfected by urologists from all over the world. Twenty years later, laparoscopic adrenalectomy *via* retroperitoneal approach was proposed, with significantly faster postoperative recovery (14). This was confirmed in later studies (15). From that standing ground, researchers shifted their focus on whether or not preserving the healthy adrenal tissue when resecting an adrenal tumor. Not surprisingly, preservation of normal functional adrenal tissue significantly reduced the risk of postoperative adrenal insufficiency (6, 16). To date, retroperitoneal laparoscopic partial adrenalectomy (RLPA) for benign lesions was accepted by the majority.

Day surgery mode has been favored by surgeons since it was proposed by the famous British surgeon Nicoll (9). Its rapid hospitality-operation-discharge mode can effectively alleviate the problem of hospital bed shortage. This is especially important in developing countries with limited medical resources. In addition, the development of minimally invasive surgery technology provides a guarantee for the operation of the day surgery mode. After minimally invasive surgery, patients suffer less injury and recover more rapidly, making administration-discharge within 24-48 hours possible. At the same time, the natural development of minimally invasive surgery also has the tendency to shift to the day surgery or outpatient surgery mode (17).

**Figure 1:**
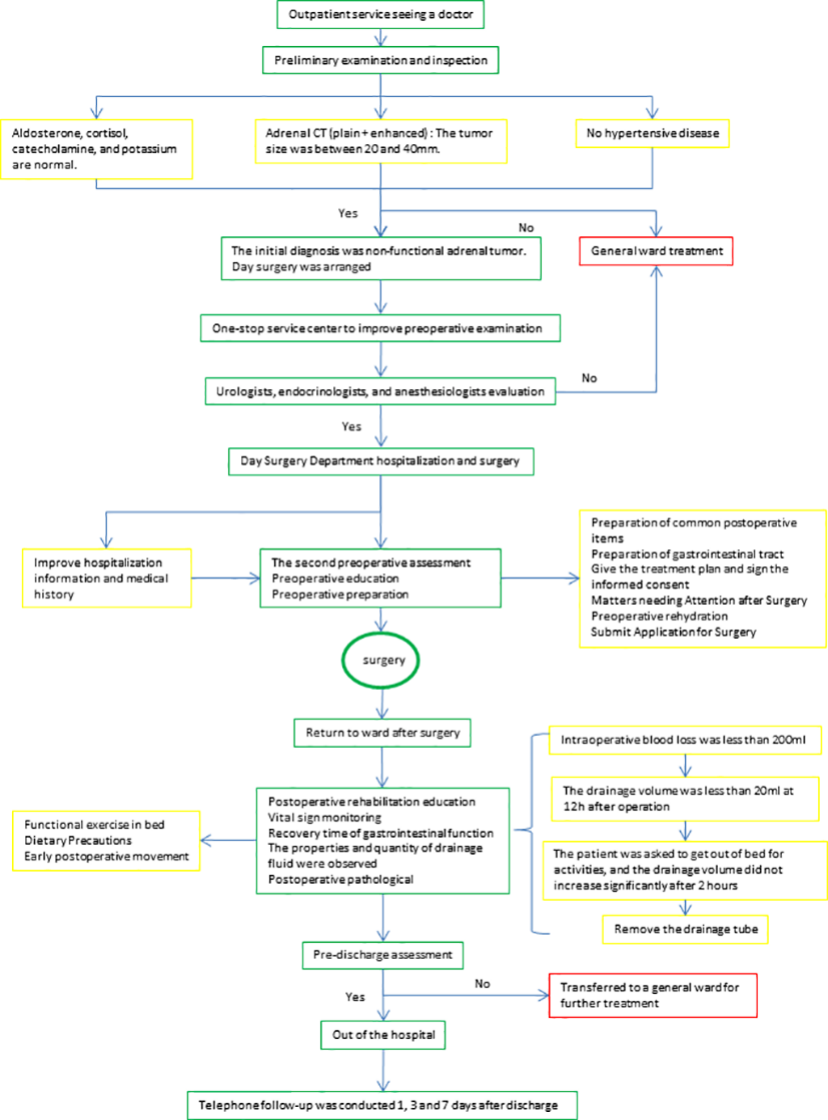
Diagnosis and treatment Procedures. After the initial selection, the patients were assigned to a one-stop service center to complete the preoperative examination. Hospitalization and surgery in the day surgery unit after assessment by the urologist, endocrinologist and anesthesiologist. After surgery, the choice of removal of the drainage tube and discharge was evaluated.

Laparoscopic adrenalectomy has been reported to be successfully performed in the day surgery or outpatient surgery mode (11, 18–20). Although partial adrenalectomy is preferable to total adrenalectomy for benign lesions, the potential risk of bleeding should be noted and hemostasis should be ensured during surgery (5). Nevertheless, a successful implantation of day surgery mode in partial adrenalectomy must be based on the premise of patient safety and surgical outcomes, which largely depend on thorough preoperative assessment and delicate surgical techniques. As such, we developed a management protocol based on the experience of our own and others (21) (**Figure 1**).

To ensure patient safety, we strictly selected patients with non-functional small tumors for RLPA in day surgery mode. For postoperative management, we tended to take personalized management on each patient based on intraoperative findings, including the amount of dissection, blood loss, vitals, and gross section view of the resected tumor. Under the pressure of Covid pandemic, we established a telephone follow-up system to detect early discomfort after discharge.

Except the 4 patients who were transferred to the general ward for extended observation and management, other patients were all discharged within 48 hours. During the postoperative telephone follow-up, there were no complaints or discomfort from any of the patients. This is similar to the results reported by Mohammad (12) for laparoscopic adrenalectomy, suggesting that the day surgery mode of RLPA is safe.

Another advantage of RLPA in the day surgery mode lies in the hospitalization cost. The cost of hospitalization for a patient admitted in a general ward is around 33,498.3 RMB¥ (counted from patients administered during the same period of time), which is almost 40% higher than the average of 24,168.4 RMB¥ in our group of patients. The average length of stay for a patient in a general ward is 7 days, which is much longer than our group of patients as well.

The major limitations of our study were its nonrandomized design and relatively small sample size. Further studies are required to establish the safety and outcomes of RLPA in day surgery mode.

## Conclusions

Retroperitoneal laparoscopic partial adrenalectomy for 20-40 mm nonfunctional unilateral adrenal tumors in the day surgery mode seems to be a safe strategy, that significantly reduce the cost and time of hospitalization.

## Data availability statement

The original contributions presented in the study are included in the article/**Supplementary Material**. Further inquiries can be directed to the corresponding authors.

## Ethics statement

The studies involving human participants were reviewed and approved by the Ethical Committee of The First Affiliated Hospital of Nanchang University. The patients/participants provided their written informed consent to participate in this study.

## Author contributions

Conception and design: XCZ and GW. Surgeons: GW and HX. Acquisition of data: WL and YY. Preparation of tools: XPZ and ZG. Analysis and interpretation of data: XL and YY. Drafting of the manuscript and statistical analysis: XL and XCZ. Critical revision: BF and XPZ. Obtaining funding: XCZ and HX. All authors contributed to the article and approved the submitted version.
